# Bezafibrate-associated rhabdomyolysis in a patient with diabetic kidney disease: A case report and successful transition to tafolecimab

**DOI:** 10.1097/MD.0000000000049269

**Published:** 2026-06-19

**Authors:** Yiping Zhang, Jinrong Wang, Xiaohong Xie, Wei Gu

**Affiliations:** a Department of Internal Medicine, Hebei Medical University, Shijiazhuang, Hebei, China; b Department of Endocrinology, Harrison International Peace Hospital, Hengshui, Hebei, China; c Department of Critical Care Medicine, Harrison International Peace Hospital, Hengshui, Hebei, China.

**Keywords:** bezafibrate, chronic kidney disease, dyslipidemia, rhabdomyolysis, tafolecimab

## Abstract

**Rationale:**

Fibrates like bezafibrate are renally excreted and can cause rhabdomyolysis in chronic kidney disease (CKD). This report describes bezafibrate-associated rhabdomyolysis in a patient with diabetic kidney disease and successful transition to tafolecimab.

**Patient concerns:**

Ten days after self-initiating bezafibrate (0.2 g thrice daily), a 71-year-old female patient with diabetes, hypertension, CKD, and dyslipidemia complained of fatigue, sweating, and myalgia. Tests revealed hypokalemia, renal failure (creatinine 329.3 μmol/L), and increased creatine kinase (4865 U/L).

**Diagnoses:**

After ruling out other causes, a peak creatine kinase of 6419 U/L (38× upper normal limit) and myalgia were used to diagnose bezafibrate-associated rhabdomyolysis.

**Interventions:**

The use of bezafibrate was stopped. Hepatoprotection, renal protection, bicarbonate, potassium correction, and eventually a switch to subcutaneous tafolecimab 150 mg every 2 weeks were all part of the supportive care.

**Outcomes:**

Myalgia went away in 6 days, and by the time of discharge, creatine kinase had returned to normal from 6419 to 54 U/L. Following tafolecimab, low-density lipoprotein cholesterol (4.18 to 2.93 mmol/L) was lower, and muscle enzymes were normal.

**Lessons:**

Bezafibrate may result in severe rhabdomyolysis in diabetic CKD, particularly when combined with nifedipine (a CYP3A4 inhibitor) and reduced renal clearance. Tafolecimab’s nonrenal metabolism and low myotoxicity make it a safe and efficient substitute.

## 1. Introduction

Managing dyslipidemia in patients with chronic kidney disease, particularly those with diabetic kidney disease, presents a significant clinical challenge due to an elevated risk of both atherosclerotic cardiovascular disease and adverse drug reactions. Fibrates, such as bezafibrate, are commonly prescribed for hypertriglyceridemia. However, they are primarily renally excreted, and their use in chronic kidney disease is associated with a well-documented, though relatively rare, risk of severe myotoxicity, including rhabdomyolysis. This risk is potentiated by drug interactions, such as with cytochrome P450 3A4 inhibitors. For patients who develop muscle-related adverse events to both statins and fibrates, alternative safe and effective lipid-lowering strategies are urgently needed. Proprotein convertase subtilisin/kexin type 9 monoclonal antibodies, like tafolecimab, offer a promising alternative due to their nonrenal route of elimination and minimal muscle side effects. This case report describes the occurrence of bezafibrate-associated rhabdomyolysis in a patient with advanced diabetic kidney disease and details the successful transition to tafolecimab, highlighting a critical therapeutic shift in a high-risk population.

## 2. Case presentation

A 71-year-old female patient was admitted with a 7-year history of hyperglycemia and more than 10 days of intermittent fatigue and profuse sweating. The patient experienced polyuria, polyphagia, and polydipsia 7 years ago. Diabetes mellitus was diagnosed after repeated venous fasting blood glucose readings ranging from 8 to 10 mmol/L. Treatment commenced with subcutaneous injections of insulin aspart before each meal (specific dosage unknown). The glucose-lowering regimen was adjusted multiple times due to poor glycaemic control. Her most recent antihyperglycemic regimen comprised glucose-lowering therapy with insulin glargine 18 U subcutaneously before dinner and dapagliflozin 10 mg orally once daily, and premeal recombinant human insulin (dose-titrated from 18–22 U based on blood glucose levels). Her self-monitored blood glucose levels typically ranged from 6 to 9 mmol/L fasting to 12 to 13 mmol/L postprandially. One year ago, the patient developed symmetric numbness in all 4 limbs without tingling. Ten days before admission, the patient started intermittently experiencing fatigue accompanied by profuse sweating. A fingerstick blood glucose reading of 2.8 mmol/L was recorded. She then discontinued insulin therapy, keeping only 10 mg of dapagliflozin once daily. Meanwhile, the patient started to experience generalized myalgia with fatigue, so she was transferred to the endocrinology ward for additional testing and treatment. The patient had developed multiple diabetic complications, including peripheral artery disease, peripheral neuropathy, and diabetic kidney disease (stage G5A3). Her medical history included a 12-year history of hypertension, with peak blood pressure readings of 200/110 mm Hg. Treatment comprised oral metoprolol (a beta-blocker) 25 mg once daily, valsartan hydrochlorothiazide (an angiotensin II receptor blocker/diuretic combination pill) 1 tablet once daily, and nifedipine sustained-release tablets (a calcium channel blocker) 10 mg once daily. Blood pressure was reported to be adequately controlled. A 12-year history of cerebral infarction (ischemic stroke) , with residual left-sided motor and sensory impairment. A 10-year history of dyslipidemia. Previously treated with atorvastatin for elevated total cholesterol, but discontinued due to abnormal liver enzymes and subsequently switched to Chinese patent medicine for lipid-lowering therapy. Ten days before admission, the patient self-initiated oral bezafibrate 0.2 g 3 times daily due to elevated triglycerides. She had an 8-year history of coronary atherosclerotic heart disease and old inferoposterior myocardial infarction, having undergone percutaneous coronary intervention 8 years ago and 7 years ago. She was currently taking “Isosorbide dinitrate 20 mg twice daily, Shexiang Baoxin Pills twice daily” for regular treatment. A 2-year history of pulmonary embolism with bilateral deep vein thrombosis. Currently taking oral Rivaroxaban 20 mg once daily. One year before admission, the patient underwent surgery at our hospital for “left intertrochanteric femoral fracture”; specific details are unavailable. Denies history of blood transfusion. Denies history of drug allergies. Denies history of food allergies.

Physical examination revealed: temperature: 36.3°C, pulse: 70 bpm, respiration: 20 bpm, blood pressure: 161/87 mm Hg. Patient alert and oriented, general condition fair. No significant abnormalities noted on cardiac, pulmonary, or abdominal examination. No pedal edema. Muscle strength and tone are normal in all extremities. Both feet showed symmetrically reduced pinprick pain perception in the distal feet, total loss of 10 g monofilament pressure perception (no perception at the first, third, and fifth metatarsal heads), and severe impairment of temperature perception (inability to distinguish a 5°C temperature difference). Auxiliary investigations: complete blood count, stool routine, thyroid function tests, and coagulation profile were generally within normal limits. Random glucose: 17.75 mmol/L (reference range < 7.8 mmol/L); glycated hemoglobin: 8.0 % (reference range 4.0–6.0 %). Hepatorenal function: urea 26.57 mmol/L (reference range 3.10–8.80 mmol/L), creatinine 329.3 μmol/L (reference range 44–97 μmol/L), uric acid 496 μmol/L (reference range 155–357 μmol/L). Lipid profile: total cholesterol 6.36 mmol/L (reference range 2.90–5.70 mmol/L), triglycerides 4.24 mmol/L (reference range 0.45–1.70 mmol/L), high‑density lipoprotein cholesterol 1.20 mmol/L (reference range > 1.04 mmol/L), low‑density lipoprotein cholesterol 4.18 mmol/L (reference range < 3.10 mmol/L). Cardiac enzymes: creatine kinase (CK) 4865 U/L (reference range 25–167 U/L), CK‑MB 97 U/L (reference range 0–25 U/L), lactate dehydrogenase (LDH) 336 U/L (reference range 120–250 U/L), α‑hydroxybutyrate dehydrogenase (HBDH) 313 U/L (reference range 72–182 U/L). Fasting C‑peptide: 7.5700 ng/mL (reference range 0.69–2.45 ng/mL). Arterial blood gas & electrolytes: pH 7.318 (reference range 7.350–7.450), partial pressure of carbon dioxide 34.8 mm Hg (reference range 35.0–45.0 mm Hg), standard bicarbonate 18.4 mmol/L (reference range 21.0–26.0 mmol/L), actual bicarbonate 17.3 mmol/L (reference range 21.0–27.0 mmol/L), potassium 3.00 mmol/L (reference range 3.50–5.30 mmol/L), indicating hypokalemia. Electrocardiogram findings: sinus rhythm, first‑degree atrioventricular block, nonspecific ST segment and T wave changes. The patient’s color Doppler ultrasonography of bilateral lower extremity arteries revealed uneven thickening of the intima‑media complex with multiple plaques in both lower extremity arteries, segmental stenosis (< 50 %) of the proximal left superficial femoral artery, segmental occlusion of the distal section with collateral vessel formation, and segmental stenosis (50–69 % in the mid‑section) of the right superficial femoral artery. Her color Doppler ultrasonography of the bilateral carotid, vertebral, and subclavian arteries revealed uneven thickening of the intima‑media complex with multiple plaques in both carotid arteries, no significant abnormalities in the bilateral internal jugular veins, and plaques in the bilateral subclavian arteries. Admission diagnosis: Type 2 diabetes mellitus, diabetic peripheral vascular disease, diabetic peripheral neuropathy, diabetic kidney disease (stage G5A3), chronic kidney disease, hypertension grade 3 (very high risk), dyslipidemia, sequelae of cerebral infarction, rheumatoid arthritis, bilateral lower limb deep vein thrombosis, coronary atherosclerotic heart disease, old inferoposterior myocardial infarction, post‑percutaneous coronary intervention, hyperuricemia, status post left intertrochanteric femoral fracture surgery.

Subcutaneous injections of 2 to 4 units of insulin aspart were administered preprandially as part of hypoglycemic therapy after admission. Sodium bicarbonate was given to treat metabolic acidosis based on arterial blood gas analysis. For the correction of hypokalemia, potassium chloride sustained-release tablets were taken orally twice daily for hypokalemia correction according to the electrolyte findings. Due to renal impairment, nifedipine extended-release was adjusted to 10 mg twice daily, and valsartan hydrochlorothiazide was stopped due to renal function. Due to the patient’s history of bilateral lower limb deep vein thrombosis, rivaroxaban 20 mg daily was continued. Bezafibrate was immediately discontinued due to markedly elevated muscle enzymes. On the fourth day of admission (January 9), the patient reported a marked worsening of muscle soreness. Physical examination: No edema was noted in the lower extremities. The Babinski signs were bilaterally negative. Muscle strength and tone were normal in all 4 limbs. Repeat laboratory testing showed: CK 6419 U/L, CK-MB 114 U/L, LDH 513 U/L, α-HBDH 518 U/L. Given the patient’s chronic kidney disease, rhabdomyolysis was strongly suspected. Treatment was initiated with: Niaoduqing granules (5 g, 4 times daily); bicyclol tablets (25 mg orally 3 times daily) for hepatoprotection with liver enzyme reduction and renal protection. Six days after discontinuing bezafibrate (January 11), the patient’s fatigue and myalgia resolved. Follow-up tests showed: CK 1265 U/L, CK-MB 25 U/L, LDH 504 U/L, α-HBDH 513 U/L, and potassium 3.88 mmol/L, representing marked improvement. The patient was discharged on January 20 after her condition stabilized. Reexamination before hospital discharge showed: CK 54 U/L, CK-MB 12 U/L, LDH 254 U/L, and α-HBDH 217 U/L. Given the patient’s history of elevated muscle enzymes with both statin and fibrate therapy, combined with a history of macrovascular disease, the lipid-lowering regimen was changed to tafolecimab 150 mg subcutaneously every 2 weeks. Nineteen days after discharge (February 8), outpatient follow-up showed that muscle enzymes remained unremarkable, and lipid levels had decreased compared to previous results (see Tables 1 and 2).

**Table 1:** Muscle enzyme and renal function levels during treatment.

	CK (25–167 U/L)	CK-MB (0–25 U/L)	LDH (120–250 U/L)	α-HBDH (72–182 U/L)	SCr (41–81 μmol/L)
Jan 6, 2025	4865	97	336	313	329
Jan 8, 2025	6419	114	513	518	278
Jan 11, 2025	1265	25	504	513	182
Jan 20, 2025	54	12	254	217	151
Feb 8, 2025	48	14	175	145	130
Aug 23, 2025	43	19	153	176	117

CK = creatine kinase, CK-MB = creatine kinase isoenzyme MB, HBDH = hydroxybutyrate dehydrogenase, LDH = lactate dehydrogenase, SCr = serum creatinine.

**Table 2:** Changes in blood lipid levels during treatment.

	TC (2.90–5.70 mmol/L)	TG (0.45–1.70 mmol/L)	HDLC (> 1.04 mmol/L)	LDLC (< 3.37 mmol/L)
2025–1-06	6.36	4.24	1.20	4.18
2025–1-20	8.11	11.01	1.80	4.22
2025–3-24	5.87	6.55	1.10	3.48
2025–8-23	4.87	3.48	1.11	2.93

HDLD = high-density lipoprotein cholesterol, LDLC = low-density lipoprotein cholesterol, TC = total cholesterol, TG = triglycerides.

## 3. Discussion and conclusions

Rhabdomyolysis is a relatively uncommon disorder affecting muscle tissue. Its typical clinical manifestations include altered urine color, marked muscle weakness, muscle atrophy in affected areas, unexplained fatigue, muscle rigidity or tenderness, and may be accompanied by complications such as electrolyte imbalance and acute kidney injury.^[1]^ The diagnostic criteria for this condition primarily comprise the following components: a relevant history of conditions predisposing to rhabdomyolysis, along with clinical manifestations such as muscle weakness and myalgia; CK levels elevated to more than 10 times the upper limit of normal; detection of myoglobin in the serum (myoglobinemia) or urine (myoglobinuria); findings of myogenic damage on electromyography, and the presence of nonspecific inflammatory changes on muscle biopsy. Fulfillment of the first 3 criteria is sufficient for diagnosis, while the fourth is reserved for differential diagnosis.^[2]^ The pathogenesis of this disease can be categorized into hereditary factors and acquired factors. Hereditary factors include glycogenolytic enzyme deficiency disorders (e.g., McArdle disease), lipid metabolism disorders (e.g., carnitine deficiency), and idiopathic rhabdomyolysis (e.g., autosomal dominant myoglobinuria). Acquired factors include alcohol or drug intoxication (e.g., statins and anesthetics), high-intensity exercise, direct muscle trauma, ischemic injury, metabolic disorders, and immune system diseases.^[3]^

In this case, the patient had no history of common precipitating factors such as high-intensity physical activity or muscle contusion. Although mild hypokalemia was present upon admission, potassium levels rapidly returned to normal following potassium supplementation, thus ruling out rhabdomyolysis caused by hypokalemia or other electrolyte disturbances. The patient had a 10-year history of dyslipidemia. After 10 days of oral bezafibrate treatment, she developed limb myalgia with fatigue, with CK peaking at 38 times the upper limit of normal. Clinical symptoms gradually resolved upon discontinuation of bezafibrate, and CK levels essentially normalized. It should be noted that although bezafibrate has been documented to induce rhabdomyolysis^[4–7]^ reports of such cases remain limited compared to statins. Further analysis of concomitant medications revealed no reports of related myotoxicity for isosorbide dinitrate, Shexiang Baoxin pills, rivaroxaban, or potassium chloride sustained-release tablets. Thus, bezafibrate is strongly implicated as the primary etiologic agent of rhabdomyolysis in this patient.

Previous studies have indicated that statin-associated rhabdomyolysis occurs more readily in patients with chronic kidney disease, closely linked to the drug’s pharmacokinetic properties: The half-life of bezafibrate is approximately 20 hours, with 80 to 90% of it excreted via the kidneys. In plasma, 99% of the drug is protein-bound, leaving only 1% in the free state.^[8]^ The clearance of the drug was significantly reduced in patients whose renal function is impaired, making the drug easier to accumulate within the body and potentially leading to rhabdomyolysis.^[9]^ An analysis by Wu et al^[10]^ of 76 cases of fibrate-associated rhabdomyolysis revealed that 21% of patients had chronic kidney disease before the initiation of fibrate therapy, 31% had a history of hypertension before the initiation of fibrate therapy, and 27% had a history of diabetes mellitus before the initiation of fibrate therapy. In this case, the patient had a 7-year history of diabetes mellitus and a 12-year history of hypertension, with diabetic kidney disease (stage G5A3) at admission. The patient was also on long-term nifedipine for hypertension. As both bezafibrate and nifedipine are metabolized by cytochrome P450 3A4, and nifedipine inhibits this enzyme, their concomitant use may have impaired the metabolism of bezafibrate. This could lead to increased plasma concentrations of bezafibrate and a consequently elevated risk of rhabdomyolysis.^[11]^ In summary, the onset in this case may be related to the patient’s chronic kidney disease and the drug interaction with nifedipine.

Tafolecimab is the first novel proprotein convertase subtilisin/kexin type 9 inhibitor lipid-lowering drug developed in China.^[12]^ A meta-analysis demonstrated that 3 Phase III clinical trials of tafolecimab confirmed the drug’s ability to significantly reduce low-density lipoprotein cholesterol levels in patients with heterozygous familial hypercholesterolemia and nonfamilial hypercholesterolemia at high cardiovascular risk, with no serious adverse reactions observed.^[13]^ As a monoclonal antibody, tafolecimab is primarily metabolized through systemic proteolytic degradation into amino acids and peptides, with its metabolism not dependent on the liver and kidneys for elimination.^[14]^ Consequently, for patients with chronic kidney disease requiring intensive lipid-lowering therapy, tafolecimab represents a safe and effective option supported by evidence-based data. Consequently, we subsequently selected tafolecimab for lipid-lowering therapy. The patient’s lipid levels decreased steadily without elevated muscle enzymes. According to a study by Qi et al^[15]^ a large-scale randomized, double-blind, placebo-controlled phase 3 study in Chinese patients at high or very high cardiovascular risk with hypercholesterolemia, tafolecimab demonstrated robust lipid-lowering efficacy, reducing low-density lipoprotein cholesterol by a least squares mean of 68.9% from baseline at week 12 (*P* < .0001 vs placebo). Notably, the study included a substantial proportion of patients with chronic kidney disease (34.1% in the tafolecimab group), and the low-density lipoprotein cholesterol‑lowering effect was consistent across patients with concomitant diseases, including chronic kidney disease, without an increase in muscle‑related adverse events (muscle events occurred in only 1.0% of tafolecimab‑treated patients). These results lend credence to tafolecimab’s potential to improve atherosclerosis and lower the risk of major adverse cardiovascular events in this high-risk group. In the present case, following treatment with tafolecimab, the patient’s low-density lipoprotein cholesterol level decreased from 4.18 to 2.93, which may delay the progression of atherosclerosis to some extent. In the present case, this therapeutic strategy is expected to favorably influence the long-term clinical trajectory.

Given the potential of bezafibrate to induce rhabdomyolysis in patients with chronic kidney disease, vigilant clinical management is warranted. This includes rigorous renal function monitoring and comprehensive patient education. Bezafibrate should be promptly discontinued, and appropriate intervention initiated if unexplained myalgia or elevated muscle enzymes occur. For patients with dyslipidemia, chronic kidney disease, and a history of atherosclerotic cardiovascular disease, tafolecimab offers a favorable therapeutic alternative due to its more favorable safety profile regarding muscle-related adverse events.

## Author contributions

**Conceptualization:** Wei Gu.

**Data curation:** Yiping Zhang, Jinrong Wang.

**Formal analysis:** Yiping Zhang.

**Methodology:** Wei Gu, Xiaohong Xie.

**Project administration:** Jinrong Wang.

**Resources:** Jinrong Wang.

**Supervision:** Wei Gu.

**Validation:** Yiping Zhang, Wei Gu.

**Writing – original draft:** Yiping Zhang, Jinrong Wang, Xiaohong Xie.

**Writing – review & editing:** Wei Gu.
